# LR-AKAP: A Lightweight and Robust Security Protocol for Smart Home Environments

**DOI:** 10.3390/s22186902

**Published:** 2022-09-13

**Authors:** Rana Muhammad Abdul Haseeb-ur-rehman, Misbah Liaqat, Azana Hafizah Mohd Aman, Abdulwahab Ali Almazroi, Mohammad Kamrul Hasan, Zeeshan Ali, Rana Liaqat Ali

**Affiliations:** 1 Faculty of Information Science and Technology, University Kebangsaan Malaysia, Bangi 43600, Malaysia; 2 Department of Information Technology, College of Computing and Information Technology, University of Jeddah, Khulais 21959, Saudi Arabia; 3 Department of Computer Science and Software Engineering, International Islamic University Islamabad, Islamabad 44000, Pakistan; 4 Department of Physics, COMSATS University Islamabad, Islamabad 45550, Pakistan

**Keywords:** Internet-of-Things (IoT), automation, sensors, smart home, authentication, protocols, session key, stolen verifier, impersonation, security, AVISPA

## Abstract

For the betterment of human life, smart Internet of Things (IoT)-based systems are needed for the new era. IoT is evolving swiftly for its applications in the smart environment, including smart airports, smart buildings, smart manufacturing, smart homes, etc. A smart home environment includes resource-constrained devices that are interlinked, monitored, controlled, and analyzed with the help of the Internet. In a distributed smart environment, devices with low and high computational power work together and require authenticity. Therefore, a computationally efficient and secure protocol is needed. The authentication protocol is employed to ensure that authorized smart devices communicate with the smart environment and are accessible by authorized personnel only. We have designed a novel, lightweight secure protocol for a smart home environment. The introduced novel protocol can withstand well-known attacks and is effective with respect to computation and communication complexities. Comparative, formal, and informal analyses were conducted to draw the comparison between the introduced protocol and previous state-of-the-art protocols.

## 1. Introduction

Human life is enviable. Science and technology play a vital role in providing safety to human life by developing safer devices using smarter technology. [1,2]. The advances in wireless and sensor technology have played an indispensable role in the rapid adaption of the smart home as an interesting new paradigm of the Internet of Things (IoT) [3,4,5,6,7,8]. This results in gaining a considerable amount of interest from both industry and academic fields alike [9].

A smart-home environment is used to assist individuals in various tasks by appending automation to them, like a smart meter, which can help users by showing the real-time consumption of power, voltage, and load [10,11] along with other interconnected devices. These devices can be various sensors, like a sensor to measure humidity, temperature, occupancy, and even to monitor the equipment. Multiple services can be provided by employing these services, such as turning on the air conditioner (AC) when an individual enters the room or turning on the lights when a person opens the door, and carbon dioxide (CO_2_) sensors can be employed to monitor the environment and to trigger automatic responses to energy saving [12,13]. In addition, these various smart gadgets can be deployed to oversee the health of the elderly [14]. Smart-home devices can also be accessed through a user’s device, such as a smartphone, without the restriction of place or time.

An innovative irrigation system may assist in conserving tens of dollars each year in addition to thousands of gallons of water. Cyber Rain [15], for example, allows observing and controlling the system from any Internet-connected device and can be customized to fit any size yard. By analyzing the weather prediction for you and modifying the watering plan automatically when rain is noticed, systems like Cyber Rain save the Earth’s limited supply of freshwater. According to the company’s website, users of Cyber Rain reportedly consume 35% less water on average.

Linked feeders provide automatic pet care. Using linked timers, you may water your yard and inside plants. There are many different types of kitchen equipment useable, such as smart coffee machines that can mix a fresh cup automatically at a set time, smart refrigerators that keep track of expiration dates, generate grocery lists, or even create ingredients using ingredients already in the house, toasters, and delayed cookers, and washing machines and dryers for the laundry room [16].

A wide range of applications, from business to government and military uses, may be realized with secure and continuous user access to designated smart home appliances [17]. Due to the sensitive nature of the operations and inherited vulnerabilities of the public channel, such as real-time data manipulation, clogging, replay, jamming, etc., the use of these smart home appliances for such applications is otherwise regarded as problematic [18,19]. Although various security schemes for the smart-home environment have recently been developed, many of these schemes have turned out to be insecure or unworkable. Consequently, the current demand is for an authentication system that can guarantee both security and privacy. To support the automatic security proof of the suggested method, we used a well-known automated program, AVISPA. The research showed that the suggested system could withstand known assaults and offered excellent balance between security and effectiveness [20,21,22,23].

**Figure 1:**
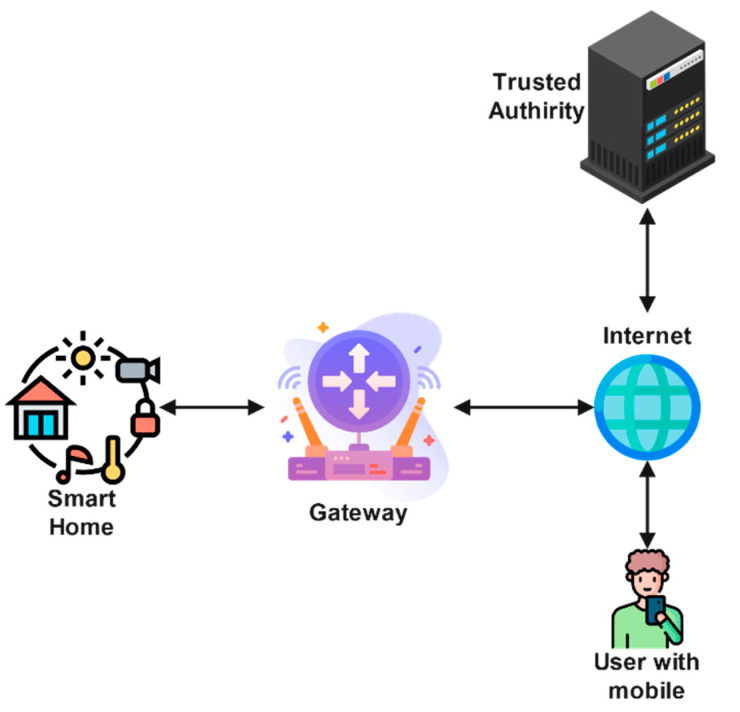
Smart home environment architecture diagram (adapted from [12]).

A typical architecture for a smart home is presented in Figure 1. The network model comprises four components that are (i) the end user ($U_{i}$), (ii) a trusted authority (TA), (iii) a gateway node (GWN), and (iv) smart devices (SD). The SDs have limited resources and can be used for various tasks such as controlling the temperature, turning on lights [20], etc. The GWN is used as an access point and has enough computation power; it acts as a gateway for U and SD. The TA is trusted by all the parties involved in the communication. The responsibilities he TA include (i) system parameters’ generation and assignment, (ii) smart device and user registration, and (iii) key maintenance and management.

This study is arranged as follows: Section 2 presents the motivation and contribution of our research. Section 3 shortly investigates the existing literature. Section 4 presents our proposed protocol. Moreover, Section 5 discusses the security analysis. Section 6 presents the performance analysis. In Section 7, the whole work is summed up.

## 2. Motivations and Contributions

This section concisely presents the primary contributions of this study:

1. We suggested an authentication protocol based on a symmetric key to protect the user–smart device connection. The system was created using a mobile device, biometrics, and a password.
2. The security of the proposed work was analyzed by using an automated security tool AVISPA.
3. The analysis showed that the suggested system could withstand known attacks and offered an excellent balance between security and effectiveness.
4. The computation cost of the proposed protocol was less than that of the protocols with the smart devices as they were resource-constrained and had limited computational power.
5. The proposed protocol evaded the clock synchronization issue in timestamp-based two-way authentication protocols.
6. Our presented protocol was compared to others in a similar field, showing that the proposed protocol was better in terms of security and performance analysis.

## 3. Related Work

Recently, various protocols have been introduced to provide security in smart home environments [24,25]. Every protocol has its strengths and weaknesses. Sciancalepore et al. [26] used ECQV and ECDH certificates to accomplish authentication. However, two distinct keys are required to run the scheme, and the efficiency of the key creation function relies on the key derivation function (KDF). Any problem in the KDF may result in a connection interruption between entities. All the keys are generated by using the master key; hence, disclosure of the master key may cause forward secrecy [27]. In [28], the authors present an authentication protocol for an IoT-enabled smart home. The authors state that their protocol is secure and lightweight against numerous attacks.

However, Ref. [27] found that their protocol lacks confidentiality and resistance against a Denial-of-Service attack (DoS) and some known key attacks. Additionally, the communication and computation costs are very high, which is not recommended for a resource-limited environment such as a smart-home environment. Dey and Hossain [29] presented a protocol using a new security model for smart homes. Gaba et al. [27] said that their protocol is efficient in computation and communication cost lacks message freshness and cannot resist a known-key attack.

Kumar et al. [30] introduced a new authentication mechanism for an SH environment. In this scheme, the SK is created by utilizing a small token to achieve authentication and theusingntity. This protocol is secure against various well-known attacks and lightweight in terms of computation and communication costs. However, after analysis, it is evident that their protocol is vulnerable to time synchronization attacks, replay attacks, does not provide anonymity, and has unlink ability issues. Kumar et al. [31] presented a new improved protocol in which the session key is renewed continuously to resist replay attacks. However, their protocol’s computation and communication costs are still very high. Gope and Sikdar [32] introduced a new protocol and claim that it is secure against impersonation, traceability, and physical attacks. However, due to enormous use of hash operations and high communication complexities, their protocol is not recommended for smart-home environments.

Wazid et al. [33] presented a protocol for smart homes. In this study, the use of an XOR operation one-way hand function makes it accessible for smart sensors. In contrast, their scheme is susceptible to a stolen verifier attack because verifier table is stored on the GWN and is vulnerable to a synchronization attack because a time stamp is employed to stop a replay attack. Shuai et al. [34] presented a protocol using ECC for a smart-home environment. They eliminated the need of a verifier table for authentication. However, their protocol incurs high communication and computation costs, which is not recommended in a smart-home environment. Banerjee et al. [23] also proposed a scheme to secure smart-home environments. They stated that their scheme can bear well-known attacks but, in this study, it is shown that their scheme is prone to a stolen gateway node attack, a stolen verifier attack, a gateway node impersonation attack towards a smart device, a broadcast issue which causes inefficient resource utilization, a smart device impersonation and a parallel session attack, and a gateway node impersonation attack towards the user. In this study, a three-factor-based enhanced scheme is introduced to overcome the shortfalls. 

## 4. Proposed Protocol

**Figure 2:**
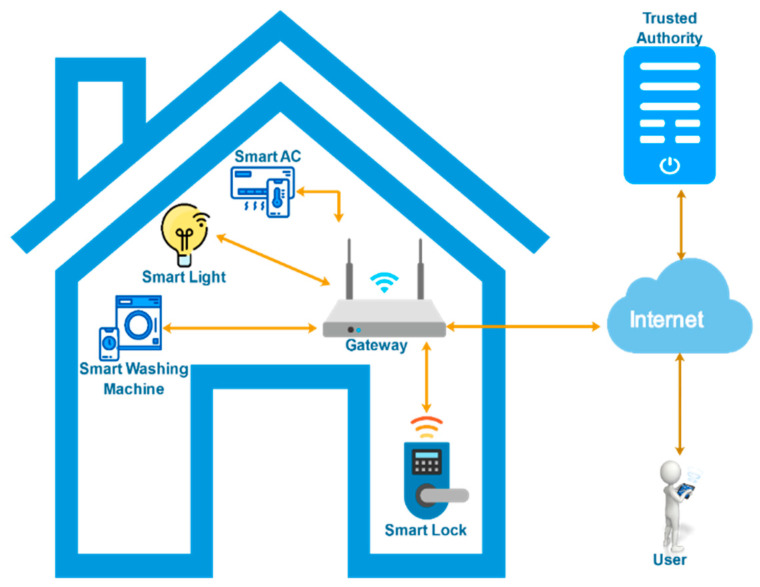
Proposed architecture for a smart-home environment.

A lightweight and robust security protocol for smart-home environments (LR-AKAP) is presented, which not only withstands well-known active and passive attacks, but also attains the required functional qualities. The proposed protocol includes six processes: (i) the initialization process, (ii) the smart device enrolment process, (iii) the gateway node enrolment process, (iv) the user enrolment process, (v) the login and authentication process, and (vi) the password and biometric update process. Various notations used in this paper are listed in Table 1. The proposed architecture of the scheme is depicted in Figure 2.

### 4.1. Assumptions

The following are the assumptions which are taken into consideration to design the introduced protocol:

1. The GWN is trusted and there is no energy restriction. Nevertheless, the sensor nodes (smart devices) are powered by batteries, and they have very limited resources [35].
2. An A may inaugurate only external attacks by employing powerful devices [36].
3. The GWN is under the user’s possession, and any wicked intruder cannot control it [37].

### 4.2. Adversarial Model

1. A common adversarial model [17,18,19], Refs. [20,21,22] is considered in this article, where the adversary A has the subsequent capabilities:
2. Public/open channel communication fully controlled by the TA.
3. A can detain, retransmit, and modify the old message. A can also cease or transmit a forged message.
4. A can get the data saved in a smart card through power analysis [17,22].
5. Any insider/privileged user or outsider can attempt to violate the privacy and security of the system.
6. The private key of the TA cannot be compromized.

### 4.3. Entities Involved in the Proposed Protocol

The network model is made up of four entities: smart device (*SD*) in the house, gateway node (*GWN*), end user (*U*), and trusted authoritie (*TA*). The smart devices are typically diverse and resource-constrained, and they may be utilized to allow a wide range of use cases including lighting control, surveillance systems, temperature management, and so on. The master node as a *GWN* installed in the house acts as a link between the user and smart gadgets. The users may remotely operate the various smart home gadgets based on their requirements. Others totally trust the *TA*’s processing and communication capabilities.

### 4.4. Proposed Protocol Processes

The proposed protocol is based on six phases as presented in the following subsections:

#### 4.4.1. Initialization Process

In the initialization phase, the Trusted Authority $\left(TA\right)$ selects a private key K.

#### 4.4.2. Smart Device Enrolment Process

SD picks an identity $SID_{d}$ and forwards to the TA. TA computes $S_{GS}=h(SID_{d}||K)$ and store $SID_{d}$ in its own database describe in Table 2.

#### 4.4.3. Gateway Node Enrollment Process

Given blow Table 3 GWN selects an identity $GID_{w}$ and sends it to TA. TA computes $N_{w}=h(GID_{w}||K)$ and stores $GID_{w}$ in its own database and sends $N_{w}$ to the GWN, which it stores in its own memory.

#### 4.4.4. User Enrollment Process

The subsequent procedure is adopted to register a user with the system in Table 4:

Step 1. U picks $ID_{u}$ and $PW_{u}$ and computes $HID_{u}=h\left(ID_{u}\right)$ and sends $HID_{u}$ to the TA.
Step 2. TA generates $R_{t1}$, $PID_{u}\in Z_{p}$ and calculates $A_{u}=h\left(HID_{u}\left|\left|R_{t1}\right|\right|K\right)$. Save $\{PID_{u},\left\{A_{u}{\}}_{E_{k}}\right\}$ tuple in own database. Send $A_{u}$, $PID_{u}$ to U through private channel.
Step 3. On getting $MSG_{2}$, U computes $\left(\sigma_{u},\tau_{u}\right)=Gen\left(Bio_{u}\right)$, $A_{u}^{*}=A_{u}⊕\sigma_{u}⊕PW_{u}$, $V_{u}=h\left(ID_{u}\left|\left|PW_{u}\right|\right|\sigma_{u}\right)⊕\sigma_{u}$, $PID_{u}^{*}=PID_{u}⊕\sigma_{u}$. SC replaces $A_{u},PID_{u}$ with $A_{u}^{*},V_{u},h\left(.\right),Gen\left(.\right),Rep\left(.\right),\tau_{u},t$ in SC.

#### 4.4.5. Login and Authentication Process

The login and authentication phase describe in Table 5 includes the following steps:

Step 1. Insert SC and input $ID_{u},PW_{u},Bio_{u}$. Calculate $\sigma_{u}=Rep\left(Bio_{u},\tau_{u}\right)$ and check whether $V_{u}⊕\sigma_{u}\overset{?}{=}h\left(ID_{u}\left|\left|PW_{u}\right|\right|\sigma_{u}\right)$; if true, further compute $A_{u}=A_{u}^{*}⊕\sigma_{u}⊕PW_{u}$. SC chooses $R_{u}\in Z_{p}$ and $T_{u}$ and computes $A_{u}^{**}=h(A_{u}||T_{u})$, $PID_{u}=PID_{u}^{*}⊕\sigma_{u}$, $SID_{d}^{*}=SID_{d}⊕A_{u}⊕R_{u}$, $R_{u}^{*}=R_{u}⊕A_{u}$. Sends $MSG_{1}=\langle R_{u}^{*},SID_{d}^{*},PID_{u},A_{u}^{**},T_{u}\rangle$ to TA by public channel.

Step 2. First of all, TA verifies the timeliness of the timestamp by inspecting the condition $\left|T_{u}-T_{c}\leq \delta T\right|$, if so, searches $PID_{u}$ in database, if it exists, then fetches related ${\left\{A_{u}\right\}}_{E_{k}}$ and computes $A_{u}={\left\{A_{u}\right\}}_{D_{k}}$ and checks if $A_{u}^{**}\overset{?}{=}h(A_{u}||T_{u})$, and if true, then selects $PID_{u}^{new}\in Z_{p}$ and $T_{ta}$, replaces the $PID_{u}$ with $PID_{u}^{new}$, and computes $W_{t}=h(h(SID_{d}||K)\left|\left|PID_{u}^{new}\right|\right|T_{ta})$, $V_{t}=h(N_{w}\left|\left|GID_{w}\right|\right|PID_{u}^{new}||T_{ta})$, $R_{u}=R_{u}^{*}⊕A_{u}$, $R_{u}^{**}=R_{u}⊕W_{t}$, $X_{t}=W_{t}⊕N_{w}$. Sends message $MSG_{2}=\langle R_{u}^{**},V_{t},X_{t},PID_{u}^{new},SID_{d},GID_{w},T_{ta}\rangle$ to GWN through public channel. Also sends $MSG_{3}=\langle PID_{u}^{new}\rangle$ back to U via open channel.

Step 3. GWN gets the $MSG_{2}$ from TA and checks if $\left|T_{ta}-T_{c}\leq \delta T\right|$, if true, computes $V_{t}\overset{?}{=}h(N_{w}\left|\left|GID_{w}\right|\right|PID_{u}^{new}||T_{ta})$, created timestamp $T_{w}$, and computed $W_{t}=X_{t}⊕N_{w}$, $V_{w}=h\left(SID_{d}\left|\left|W_{t}\right|\right|T_{w}\right)$. At the end, send message to the SD message $MSG_{4}=\langle R_{u}^{**},V_{w},PID_{u}^{new},SID_{d},T_{ta},T_{w}\rangle$ to SD via open/insecure channel.

Step 4. SD verifies the freshness of $MSG_{4}$ by examining the condition $\left|T_{w}-T_{c}\leq \delta T\right|$. If true, it selects $R_{d}\in Z_{p}$ and $T_{d}$, checks if $V_{w}\overset{?}{=}h\left(SID_{d}\left|\left|h\left(S_{GS}\left|\left|PID_{u}^{new}\right|\right|T_{ta}\right)\right|\right|T_{w}\right),$ if true, then computes $R_{u}=R_{u}^{**}⊕h(S_{GS}||T_{ta})$, $SK_{du}=h\left(R_{u}\left|\left|R_{d}\right|\right|PID_{u}^{new}\left|\left|SID_{d}\right|\right|T_{d}\right)$, $R_{d}^{*}=R_{u}⊕R_{d}$, $V_{d}=h(SK_{du}||T_{d})$, $X_{d}=h(W_{t}^{*}||T_{d})$. At the end, sends the message $MSG_{5}=\langle V_{d},X_{d},R_{d}^{*},T_{d}\rangle$ to GWN by public/insecure channel.

Step 5. GWN examines the freshness of the message by inspecting the condition $\left|T_{d}-T_{c}\leq \delta T\right|$, and if the condition is true, GWN selects timestamp $T_{w}^{*}$ and sends the $MSG_{6}=\langle V_{d},R_{d}^{*},T_{d},T_{w}^{*}\rangle$ to U via open channel.

Step 6. Upon getting the $MSG_{6}$, U validates message freshness through condition $\left|T_{w}^{*}-T_{c}\leq \delta T\right|$. If true, compute $PID_{u}^{**}=PID_{u}^{*}⊕PID_{u}⊕PID_{u}^{new}$, replace $PID_{u}^{*}$ with $PID_{u}^{**}$, $R_{d}=R_{u}⊕R_{d}^{*}$, $SK_{ud}=h\left(R_{u}\left|\left|R_{d}\right|\right|PID_{u}^{new}\left|\left|SID_{d}\right|\right|T_{d}\right)$. $V_{d}\overset{?}{=}h(SK_{ud}||T_{d})$, if true, the session key is saved for secure communication.

#### 4.4.6. Biometric and Password Update Process

A registered user can update a biometric/password. To do so, the user needs to login first as described in the “Login and authentication process”. After a successful login, the user will adopt the subsequent procedure to update his/her biometric/password:

Step 1. User will be prompted to enter a new password $PW_{u}^{new}$ biometric $Bio_{u}^{new}$.
Step 2. SC will compute $\left(\sigma_{u}^{new},\tau_{u}^{new}\right)=Gen\left(Bio_{u}^{new}\right)$, $A_{u}^{new}=\left(A_{u}^{*}⊕\sigma_{u}^{old}⊕PW_{u}^{old}\right)⊕\left(\sigma_{u}^{new}⊕PW_{u}^{new}\right)=A_{u}⊕\sigma_{u}^{new}⊕PW_{u}^{new}$, $V_{u}^{new}=\left(V_{u}⊕h\left(ID_{u}\left|\left|PW_{u}^{old}\right|\right|\sigma_{u}^{old}\right)⊕\sigma_{u}^{old}\right)⊕\left(h\left(ID_{u}\left|\left|PW_{u}^{new}\right|\right|\sigma_{u}^{new}\right)⊕\sigma_{u}^{new}\right)=V_{u}⊕h\left(ID_{u}\left|\left|PW_{u}^{new}\right|\right|\sigma_{u}^{new}\right)⊕\sigma_{u}^{new}$, $PID_{u}^{new}=PID_{u}^{*}⊕\sigma_{u}^{old}⊕\sigma_{u}^{new}$.
Step 3. Finally, SC will replace $A_{u}^{*},V_{u},PID_{u}^{*},\tau_{u}$ with $A_{u}^{new},V_{u}^{new},PID_{u}^{new},\tau_{u}^{new}$.

## 5. Security Analysis

In the subsequent subsection, formal and informal security analyses for our proposed protocol are performed.

### 5.1. Informal Security Analysis

The introduced protocol is secure against well-known attacks as presented in the successive subsections:

#### 5.1.1. Replay Attack

In the introduced protocol, timestamps $T_{u},T_{ta},T_{w},T_{d}$ and random numbers $R_{u},R_{d}$ are employed to ensure that the introduced protocol is secure from replay attack. Hence, the replay message will be detected if a timestamp is expired, or a random/arbitrary number is inconsistent. So, a replay attack cannot be launched upon the introduced scheme.

#### 5.1.2. Session Key Freshness Property

In the introduced protocol, a session key is constructed by some distinct identities, random numbers, and timestamps, and for each session, random numbers $R_{u},R_{d}$ are unique. Hence, a novel key for every session ensures the key’s freshness.

#### 5.1.3. User Anonymity and Untraceability

In the introduced protocol, the user identity is not shared even with the TA. Additionally, if an attacker tries to capture messages $MSG_{1},MSG_{2},MSG_{3},MSG_{4},MSG_{5},MSG_{6}$, none of these messages utter any information about the user. Furthermore, the attacker will not be able to extract from the smart card, because he/she will need a password and a biometric to extract the password, and it is also wrapped by hash a function. In addition, in each session, all the values are unique as they are composed of random numbers, and consequently, they are novel for each authentication session. Hence, an adversary will not be able to recognize any specific user or the location of any specific user. Hence, the introduced protocol ensures the anonymity and untraceability of valid user.

#### 5.1.4. Smart Card Stolen Attack

Assume an attacker steals an authorized user’s smart card or it is mishandled and discovered by an attacker, allowing him/her to get any sensitive data from the SC. An attacker can extract information $A_{u}^{*},V_{u},PID_{u}^{*},\tau_{u},t$ from the SC. But to attain any confidential information from the SC, an attacker needs the information of $ID_{u}$, $PW_{u}$, and $\sigma_{u}$, and these values are unknown to A. Hence, in the introduced protocol, a stolen SC attack is not conceivable.

#### 5.1.5. Impersonation Attack

If an attacker tries to imitate a U, GWN, or SD towards any other legal participant, to do so, a valid request messages is required. To impersonate as a U, A tries to create a login request message $MSG_{1}=R_{u}^{*},SID_{d}^{*},PID_{u},A_{u}^{**},T_{u}$. However, the attacker cannot generate a true login message because it necessitates the information of $R_{u},A_{u}$. Therefore, the introduced protocol can resist an impersonation attack.

#### 5.1.6. Man-in-the-Middle Attack

Assume an attacker seizes the message and tries to modify a login request or any other message transferring over the public channel. To do this, the attacker requires the knowledge of secret parameters as described in Section 5.1.5. Therefore, the introduced scheme can withstand this attack.

#### 5.1.7. Perfect Forward Secrecy

The shared session key in the introduced protocol includes random nonces included by both U and SD. So, if the private key K is in the knowledge of the adversary, he/she still cannot produce previously shared session keys. Hence, the introduced protocol has the feature of perfect forward secrecy.

### 5.2. AVISPA Tool Based Automated Formal Security Analysis

AVISPA, a tool relying on the Dolev–Yao threat model, was introduced by Armando et al. [38]. In this situation, the attacker can edit, transmit, and alter messages. This tool is well-accepted for assessing various security protocols. AVISPA uses a “high level protocol specification language (HLPSL)” to describe and design security protocols. The HLPSL requirements are broken down into roles. During the execution of a protocol, several roles are utilized to express the activities of a single agent. Each agent performs a specific role throughout the execution of a protocol. HLPSL’s main objective is to verify security properties including message, agent secrecy and authentication. The security protocol is considered to determine its level of security considering the predetermined objectives.

#### 5.2.1. AVISPA Model Checkers

1. **On-the-fly model checker (OFMC):** OFMC uses lazy data types to develop an efficient on-the-fly model for security protocols with limitless state spaces.
2. **Constraint-logic-based attack searcher (CL-AtSe):** The input of (CL-AtSe) is a protocol stated as a set of restrictions that help identify security protocol assaults in the form of a collection of rewriting rules (IF format).
3. **SAT-based model checker (SATMC):** Depending on the transitional state of the IF specification, creates a propositional formula. According to the propositional formula, every violation of security that might result in an attack is considered.
4. **“Tree automata-based on automatic approximations for the analysis of security protocols“ (TA4SP) model checker:** By accurately estimating the attacker’s capabilities, it exposes the protocol’s weakness and predicts its accuracy.

#### 5.2.2. AVISPA Simulation Steps

**Figure 3:**
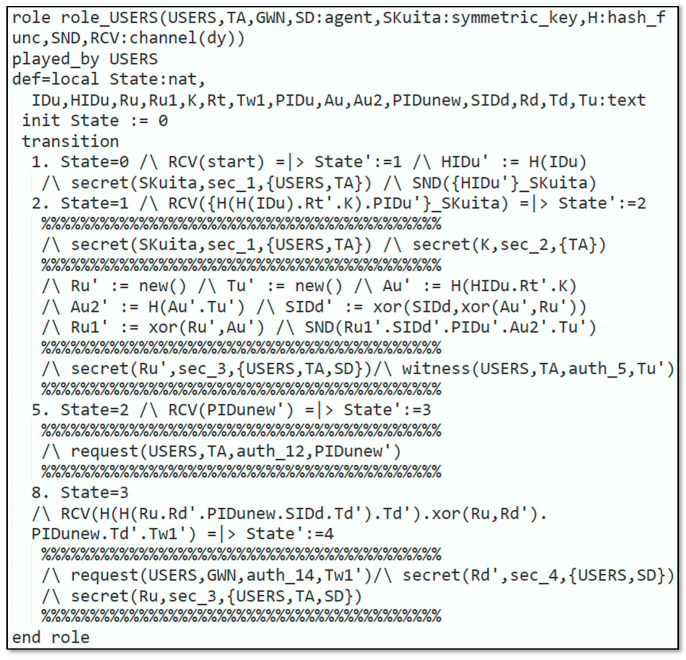
Role specification for the user.

**Figure 4:**
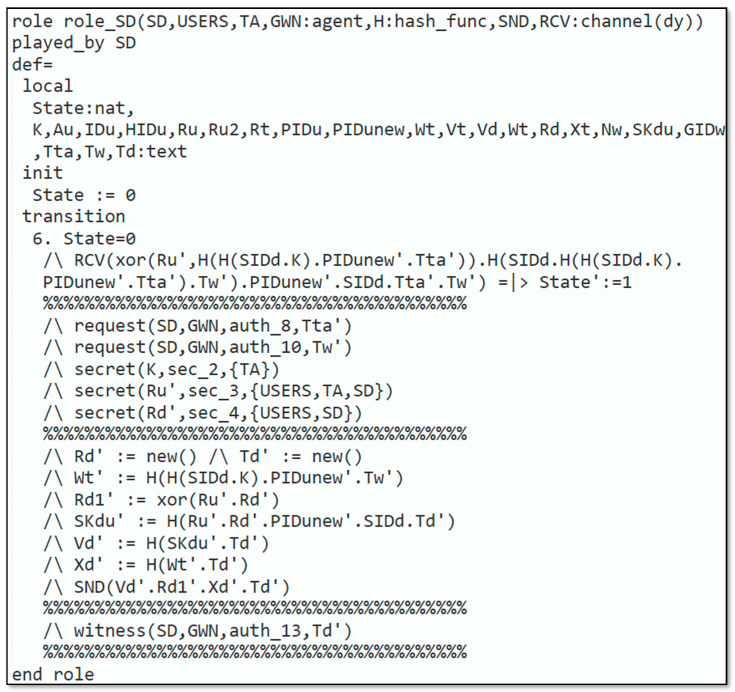
Role specification for a smart device.

**Figure 5:**
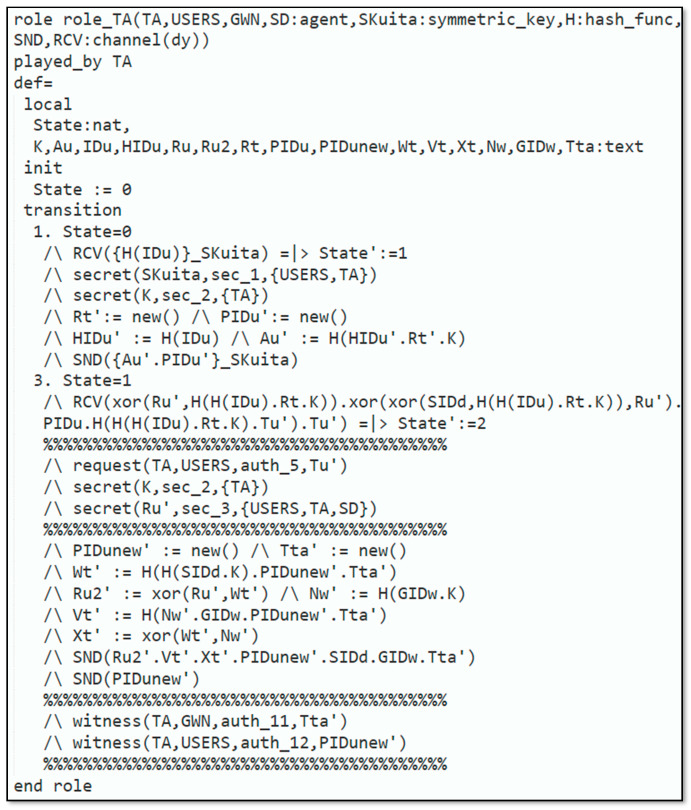
Role specification for TA.

**Figure 6:**
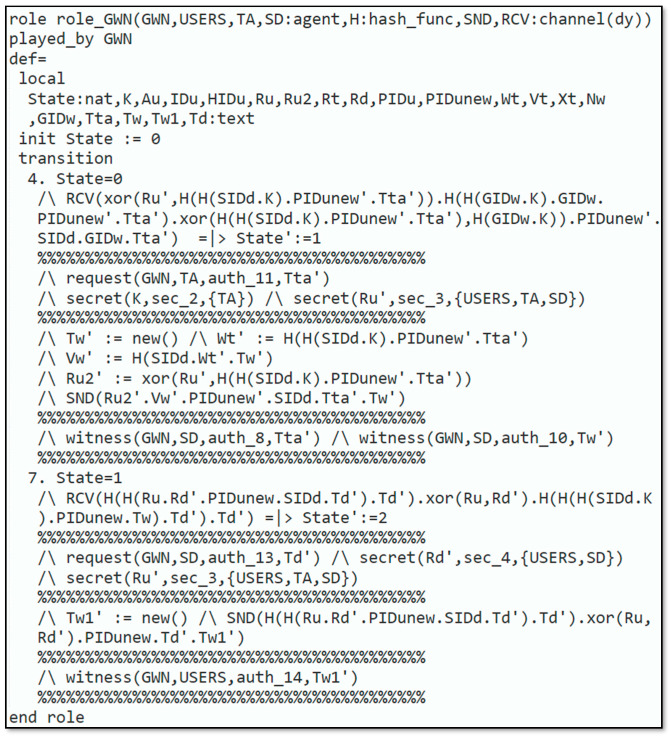
Role specification for GWN.

Automated validation of the protocol was carried out to demonstrate that the introduced protocol could resist reply and man-in-the-middle attacks. The subsequent steps were executed to simulate our protocol in AVISPA:

1. Firstly, the scheme was implemented through High-Level Protocols Specification Language (HLPSL) [33], next, the HLPSL2IF translator was employed to interpret HLPSL into Intermediate Format (IF).
2. Additionally, to declare that whether protocol was safe or not, the interpreted IF was provided in Output Format (OF). The HLPSL role specification for the user, smart device, trusted authority and gateway node are depicted in Figure 3, Figure 4, Figure 5 and Figure 6, respectively.

#### 5.2.3. Simulation Details

Setting simulated security goals is the first step in building the HLPSL script for our scheme. Our major goal was to keep certain values, such as $\left\{SK_{uita},\;K,\;R_{u},\;R_{d},\;R_{t}\right\}$, secret. Furthermore, we defined the six roles, which are: (1) the trusted authority’s role, (2) the user’s role, (3) the gateway node’s role, (4) the smart device’s role, (5) the role session that combines the basic roles (role_TA, role_USERS, role_GWN, and role_SD), (6) the role environment that incorporates numerous sessions and consists of functions and global variables and outlines the security goals of the protocol.

In Figure 3, the requirement for the role_User that is executed by the user is illustrated. In this role, the $U_{u}$ knows every agent $\left(GWN,\;SD,\;TA\right)$ and the symmetric-key $SK_{uita}$ that is joint among the $U_{u}$, TA, the hash function $H\left(.\right)$, and the Dolev–Yao dy model-based send/receive channels $\left(SND,RCV\right)$. To start the protocol, $U_{u}$ receives a start message $\left(RCV\left(start\right)\right)$ as an indication at the first state (state 0). To register, $U_{u}$ creates the identity $ID_{u}$ and retransmits $HID_{u}^{\prime }$ after being encrypted with $SK_{uita}$ to the TA. $U_{u}$ gets $H\left(H\left(HID_{u}\left|\left|R_{t}^{\prime }\right|\right|K\right)\right)$ and $PID_{u}^{\prime }$ encrypted with $SK_{uita}$ coming from the TA, and the registration process is completed. Next, $U_{u}$ generates some fresh nonces $\left\{R_{u}^{\prime },T_{u}^{\prime }\right\}$ and computes $\left\{A_{u}^{\prime },A_{u2}^{\prime },SID_{d}^{\prime }\right\}$. Next, $U_{u}$ transmits $\left\{R_{u1}^{\prime },SID_{d}^{\prime },PID_{u}^{\prime },A_{u2}^{\prime },T_{u}^{\prime }\right\}$ to TA and receives a message from the TA containing $PID_{unew}^{\prime }$. $U_{u}$ receives the message $\left\{H\left(H\left(R_{u}.R_{d}^{\prime }.PID_{unew}.SID_{d}.T_{d}^{\prime }\right).T_{d}^{\prime }\right).xor\left(R_{u},R_{d}^{\prime }\right).PID_{unew}.T_{d}^{\prime }.T_{w1}^{\prime }\right\}$ coming from the SD at the next transition. Figure 4**.** demonstrates the specification for the role_SD which is played by the SD. In this role, the SD recognizes all the agents $\left(U_{i},\;TA,\;GWN\right)$, the symmetric-key $S_{GS}$ which is shared among the SD and the TA, the hash function $H\left(.\right)$, and the Dolev–Yao-model-based send/receive channels $\left(SND,\;RSV\right)$.

Figure 5 explains the requirement for role_TA, which is executed by the TA. The TA knows every single agent $\left(U_{u},\;SD,\;GWN\right)$, the symmetric-key $SK_{uita}$, $S_{GS}$, and $N_{w}$ which are common among the $U_{u}$ and the TA, SD, and TA, and among GWN and TA, respectively. Furthermore, TA has the knowledge of the $H\left(.\right)$ and Dolev–Yao-model-based send/receive channels $\left(SND,\;RSV\right)$. The remaining part of the requirement defines the various states of the protocol implemented by the TA. Figure 6 displays the specification for role_GWN that is played by the GWN. GWN identifies all the agents $\left(U_{u},\;SD,\;TA\right)$, the symmetric-key $N_{w}$ that is shared amongst the GWN and the TA, the $H\left(.\right)$, and Dolev–Yao-model-based send/receive channels $\left(SND,\;RSV\right)$.

**Figure 7:**
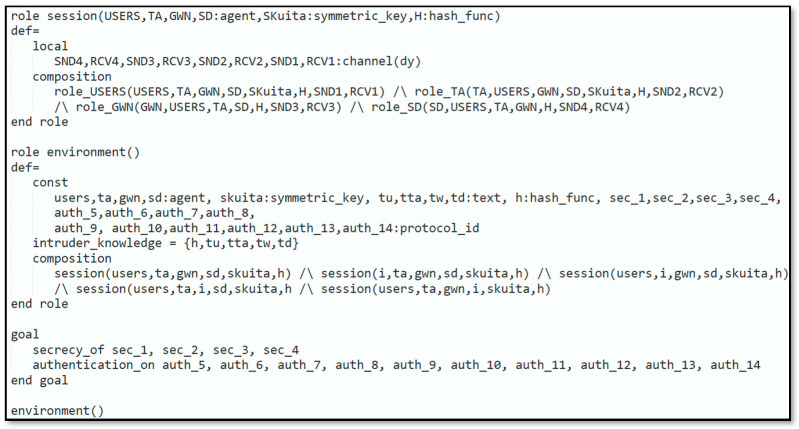
Role session, environment, and goal.

Session, environment, and goal roles’ specifications are depicted in Figure 7. In the session role, all roles (role_TA, role_USERS, role_SD, role_GWN) are combined. More than one session is started from the environment role. The constants (ta, users, gwn, sd) represents the agents $\left(TA,USERS,GWN,SD\right)$, respectively. The symmetric key shared among the TA and $U_{u}$ is presented by $SK_{uita}$. The hash function H is presented by the constant h. The applicable parameters that are supposed to be known by the intruder are defined in the intruder knowledge part. We undertake that the intruder has the knowledge of all the agents $\left(USERS,\;\;TA,\;\;GWN,\;\;SD\right)$. The simulation objectives are declared with the help of goal keywords: We are concerned about verifying the secrecy of the subsequent parameters: R_u, R_t, R_d, K.

#### 5.2.4. Simulation Result

**Figure 8:**
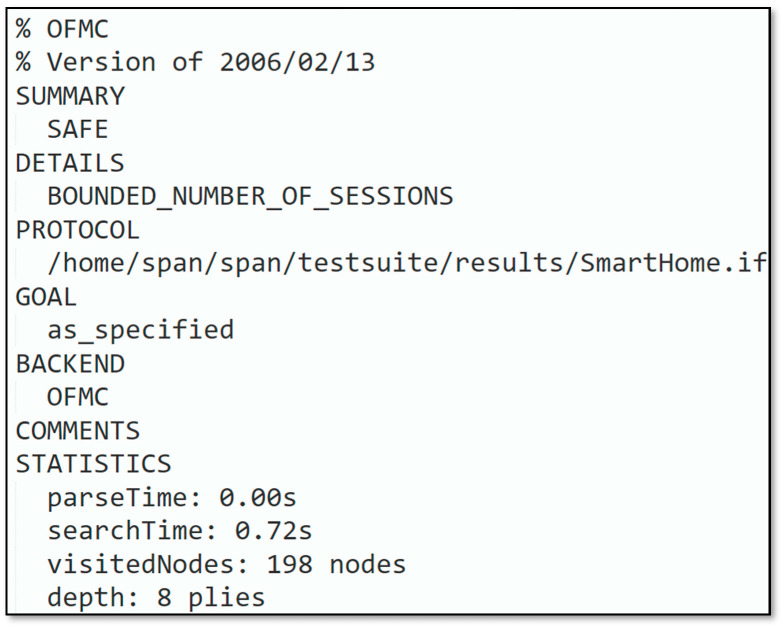
OFMC backend analysis.

**Figure 9:**
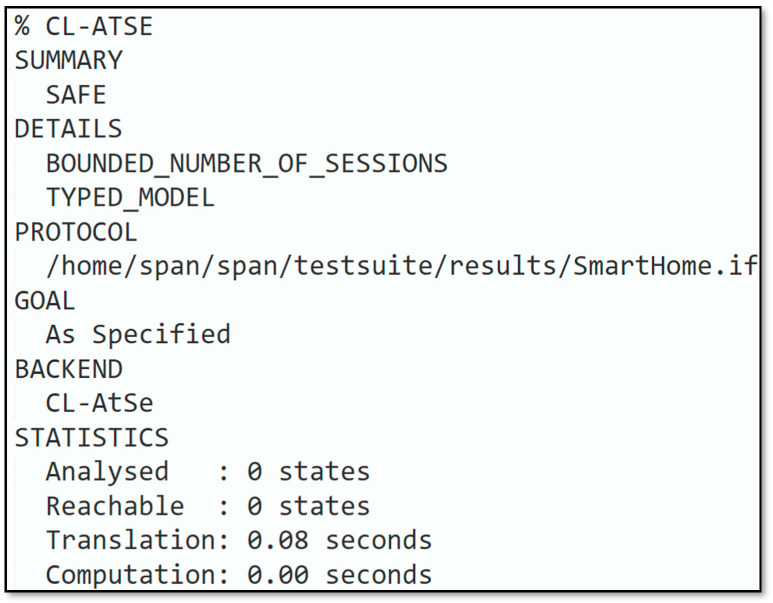
CL-AtSe backend analysis.

Simulation outcomes depicted in Figure 8 and Figure 9 revealed that the introduced protocol was according to the design specifications and could hold out against replay and man-in-the-middle attacks. Based on the AVISPA backend model checker, OFMC and CL-AtSe simulation results are presented. With depth 8 piles in 0.72 s, 198 nodes were scrutinized in the OFMC backend. The CL-AtSe backend inspected 0 states, the computation and interpretation consumed for the backend were 0.08 s and 0.00 s, respectively.

## 6. Comparative Analysis

This section provides the comparison of our proposed protocol with the existing protocols, which includes those of Banerjee et al. [23], Shuai et al. [34], Yu and Li [39], Naoui et al. [40], Fakroon et al. [41], and Dey and Hossain [29].

### 6.1. Functionality Comparison

The functionality analysis was performed with respect to the existing and proposed protocols as presented Table 6. As per comparison, the proposed protocol rendered superior security along with enhanced security features in contrast to those of [23] and other compared protocols. Here, $F_{n}$ is the $n^{\mathrm{th}}$ compared feature/security requirement, ✓ shows that the specified parameter exists in this study, and the protocol can resist an attack. Moreover, × presents whether the protocol is not able to resist an attack or lacks a specific feature, whereas the − sign shows that a certain security/feature requirement is not applicable.

### 6.2. Comparison of Communication Overhead

In Table 7, the communication expenses estimate is presented. In order to compare, the sizes of various parameters taken were as follows: identities were considered as 128 bits, a timestamp was 32 bits, random nonces were 128 bits, a hash digest was 160 bits (if SHA-1 is employed [42]), cost for ECC point was $\left(160+160\right)=320$ bits, and size of symmetric enc/decryption block was 128 bits [43,44], respectively.

**Figure 10:**
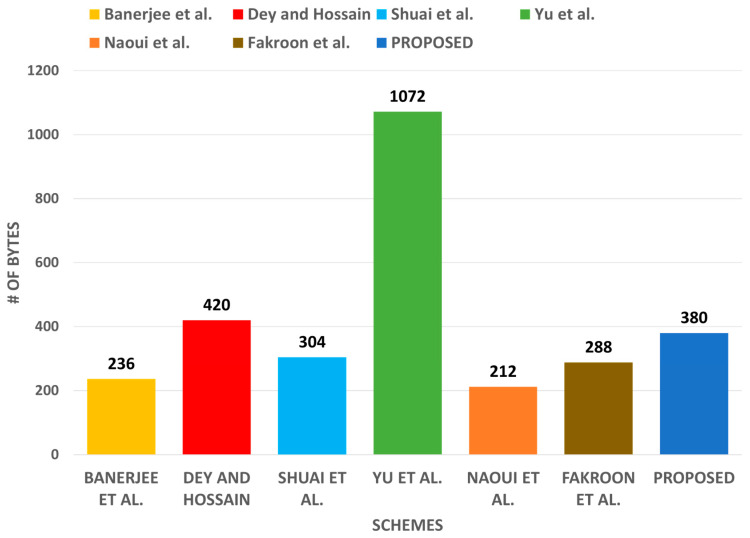
Communication cost comparison.

The communication cost of $M_{sg1}=\langle {\bar{R}}_{u}^{*},SID_{d}^{*},PID_{u},A_{u}^{**},T_{u}\rangle$ was ⟨128+128+128+160+32⟩=608 bits, $M_{sg2}=\langle R_{u}^{**},V_{t},X_{t},PID_{u}^{new},SID_{d},GID_{w},T_{ta}\rangle$ was ⟨160+160+160+128+128+128+32⟩=896 bits, $M_{sg3}=\langle PID_{u}^{new}\rangle$ was 832 bits, $M_{sg4}=\langle R_{u}^{**},V_{w},PID_{u}^{new},SID_{d},T_{ta},T_{w}\rangle$ was ⟨128+160+128+128+32+32⟩=576 bits, $M_{sg5}=\langle V_{d},X_{d},R_{d}^{*},T_{d}\rangle$ is ⟨160+160+128+32⟩=480 bits, and cost for $M_{sg6}=\langle V_{d},R_{d}^{*},T_{d},T_{w}^{*}\rangle$ was ⟨160+128+32+32=352⟩ bits. Summing all these, the total cost of communication of the introduced protocol through the login and authentication phase becomes 380 bytes. In addition, Table 7 shows that the communication cost of the introduced protocol was less than that of those in comparison [39,42] and slightly higher than that of [23,34,40,41], but this is justifiable as the introduced protocol provides better security than these other protocols do. Figure 10 also depicts the communication cost of our proposed protocol.

### 6.3. Computation Overhead Comparison

This section compares the computation cost of different protocols. Table 8 shows the approximate computation times expected for different cryptographic procedures and their notations. Based on a real-time environment, an experiment was conducted over a smartphone using MIRACL Library. The smartphone Redmi Note 8 presented as a mobile/user device with the specification of Octa-core Max 2.01 GHz processor, 4 GB RAM, MIUI version 11.0.7, and the underlying Android version was 9. For GSS, over the Ubuntu 16.0 LTS operating system, an HP EliteBook 8460P with 4 GB RAM and an Intel Core i7-2620 M 2.7 GHz processor was used. Additionally, to replicate the smart devices, a Pi3 B+ with Cortex-A53(ARMv8) 64-bit SoC @ 1.4 GHz processor and 1 GB LPDDR2 SDRAM RAM was used. The simulation results on each device are given in Table 8, which shows the approximate computation times expected for different cryptographic procedures in ms and their notations [45]. We assumed here that $T_{fe}\approx T_{ecm}$.

**Figure 11:**
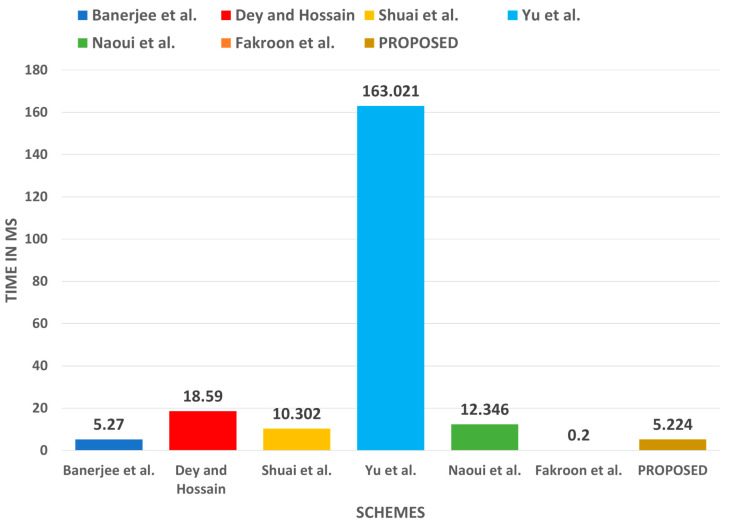
Computation cost comparison.

As represented in Table 9, the computation cost of the proposed protocol is a bit high as compared to those of [23,41]. However, it is justified when compared to the security provided by the introduced protocol, as it is shown in Table 6 that these protocols lack some security features. Figure 11 also depicts the computation cost of the introduced protocol.

## 7. Conclusions

The Internet of Things (IoT) is turning into the mainstream with each passing day. Applications of the IoT are not limited to only military or industrial use; nowadays, the IoT is employed in homes, agriculture, and even in the medical field. IoT privacy and security issues gradually increase. To subdue the privacy and security issues related to smart homes, various schemes have been introduced and claimed as robust and with anonymous authentication protocols for a smart-home environment. In this paper, we introduced a protocol to overcome the vulnerabilities found in a previous protocol. In the introduced protocol, no parameters are stored in the verifier table whose disclosure can lead to system compromise, a TA is also included to authenticate the users, and a broadcasting issue is also mitigated to improve resource utilization. Security and comparative analyses of the introduced protocol were performed to show that the introduced protocol was secure and provided better security features with low communication and computation costs. Authentication schemes for a smart-home environment should be lightweight and secure. Many schemes proposed earlier are lightweight but not secure because in achieving lightweight features they neglect security. Similarly, some schemes are more secure but not lightweight, which is undesirable for a smart-home environment. Hence, there is a need to work on both factors at the same time.

## Figures and Tables

**Table 1:** Notations guide.

Symbols	Representations
$ID_{u},PW_{u},\;Bio_{u}$	$u^{\mathrm{th}}$ users biometric and password, user’s identity
$PID_{u}$	Temporary identity of a user
$SD,SID_{d}$	Smart sensor and its identity
$GWN,GID_{w}$	Gateway node and its identity
$R_{u},R_{d}$	Random numbers
K	1024 b it-long secret key of TA
$SK_{du}\left(=SK_{ud}\right)$	Session key between U and SD
$h\left(.\right)$	Cryptographic one-way hash function
$T_{u},T_{ta},T_{w},T_{d}$	Current timestamps
$Gen\left(.\right),Rep\left(.\right)$	Fuzzy extractor probabilistic generation and deterministic reproduction function
△T	Maximum allowable transmission delay
$i\overset{?}{=}j$	Checks if i equals to j
||, ⊕	Concatenation and bitwise XOR operators
$A,I,U_{A}$	An adversary, intruder, and privileged insider

**Table 2:** Proposed smart device enrollment process.

Smart Device (SD)	Trusted Authority (TA)
$\mathrm{Selects}\;\mathrm{an}\;\mathrm{identity}\;SID_{d}$	
$\overset{MSG_{1}=\langle SID_{d}\rangle }{\to }$	
	TA computes
	$S_{GS}=h(SID_{d}||K)$
	$\mathrm{Store}\;SID_{d}$ in own database.
	$\overset{MSG_{2}=\langle S_{GS}\rangle }{\leftarrow }$
$\mathrm{Store}\;SID_{d},S_{GS}$ into memory.	

**Table 3:** Proposed gateway node enrollment process.

Gateway Node (GWN)	Trusted Authority (TA)
$\mathrm{Selects}\;\mathrm{an}\;\mathrm{identity}\;GID_{w}$	
$\overset{MSG_{1}=\langle GID_{w}\rangle }{\to }$	
	TA computes
	$N_{w}=h(GID_{w}||K)$
	$\mathrm{Stores}\;GID_{w}$ in own database.
	$\overset{MSG_{2}=\langle N_{w}\rangle }{\leftarrow }$
$\mathrm{Store}\;N_{w}$ into memory.	

**Table 4:** Proposed user enrollment process.

User (U)	Trusted Authority (TA)
$\mathrm{choose}\;ID_{u}$$\;\mathrm{and}\;PW_{u}$.	
$HID_{u}=h\left(ID_{u}\right)$	
$\overset{MSG_{1}=\langle HID_{u}\rangle }{\to }$	
	$\mathrm{Generate}\;R_{t1}$$,\;PID_{u}\in Z_{p}$.
	$\mathrm{Calculate}\;A_{u}=h\left(HID_{u}\left|\left|R_{t1}\right|\right|K\right)$.
	$\mathrm{Save}\;\{PID_{u},\left\{A_{u}{\}}_{E_{k}}\right\}$ tuple in own DB.
	$\overset{MSG_{2}=\langle A_{u},PID_{u}\rangle }{\leftarrow }$
$\left(\sigma_{u},\tau_{u}\right)=Gen\left(Bio_{u}\right)$,	
$A_{u}^{*}=A_{u}⊕\sigma_{u}⊕PW_{u}$,	
$V_{u}=h\left(ID_{u}\left|\left|PW_{u}\right|\right|\sigma_{u}\right)⊕\sigma_{u}$,	
$PID_{u}^{*}=PID_{u}⊕\sigma_{u}$.	
$\mathrm{Replace}\;A_{u},PID_{u}$ $\;\mathrm{with}\;A_{u}^{*},V_{u},PID_{u}^{*},h\left(.\right),Gen\left(.\right),Rep\left(.\right),\tau_{u},t$	
in SC.	

**Table 5:** Proposed login and authentication process.

User (U)	Trusted Authority (TA)	Gateway (GWN)	Smart Device (SD)
Insert SC, $\mathrm{Input}\;ID_{u},PW_{u},Bio_{u}$, Calculate $\sigma_{u}=Rep\left(Bio_{u},\tau_{u}\right)$, $V_{u}⊕\sigma_{u}\overset{?}{=}h\left(ID_{u}\left|\left|PW_{u}\right|\right|\sigma_{u}\right)$, $A_{u}=A_{u}^{*}⊕\sigma_{u}⊕PW_{u}$, $\;\mathrm{Choose}\;R_{u}\in Z_{p}^{*}$$\;\mathrm{and}\;T_{u}$, $A_{u}^{**}=h(A_{u}||T_{u})$, $PID_{u}=PID_{u}^{*}⊕\sigma_{u}$, $SID_{d}^{*}=SID_{d}⊕A_{u}⊕R_{u}$, $R_{u}^{*}=R_{u}⊕A_{u}$.			
$\overset{MSG_{1}=\langle R_{u}^{*},SID_{d}^{*},PID_{u},A_{u}^{**},T_{u}\rangle }{\to }$			
	$\;\mathrm{Check}\;\mathrm{if}\;\left|T_{u}-T_{c}\leq \delta T\right|$ $\;\mathrm{Search}\;PID_{u}$ in database. If exists, $\mathrm{then}\;\mathrm{fetch}\;\mathrm{related}\;{\left\{A_{u}\right\}}_{E_{k}}$, $\;A_{u}={\left\{A_{u}\right\}}_{D_{k}}$. $\;\mathrm{If}\;A_{u}^{**}\overset{?}{=}h(A_{u}||T_{u})$ true, continue.		



	$\;\mathrm{Select}\;PID_{u}^{new}\in Z_{p}$$\;\mathrm{and}\;T_{ta}$, $\;\mathrm{Replace}\;PID_{u}$$\;\mathrm{with}\;PID_{u}^{new}$ and compute $\;W_{t}=h(h(SID_{d}||K)\left|\left|PID_{u}^{new}\right|\right|T_{ta})$ $\;V_{t}=h(N_{w}\left|\left|GID_{w}\right|\right|PID_{u}^{new}||T_{ta})$, $\;R_{u}=R_{u}^{*}⊕A_{u}$, $\;R_{u}^{**}=R_{u}⊕W_{t}$, $\;X_{t}=W_{t}⊕N_{w}$. $\;\overset{MSG_{2}=\langle R_{u}^{**},V_{t},X_{t},PID_{u}^{new},SID_{d},GID_{w},T_{ta}\rangle }{\to }$ $\;\overset{MSG_{3}=\langle PID_{u}^{new}\rangle }{\leftarrow }$		






		$\;\mathrm{Check}\;\mathrm{if}\;\left|T_{ta}-T_{c}\leq \delta T\right|$ $\;V_{t}\overset{?}{=}h(N_{w}\left|\left|GID_{w}\right|\right|PID_{u}^{new}||T_{ta})$, $\;\mathrm{Create}\;\mathrm{timestamp}\;T_{w}$, $\;W_{t}=X_{t}⊕N_{w}$, $\;V_{w}=h\left(SID_{d}\left|\left|W_{t}\right|\right|T_{w}\right)$. $\;\overset{MSG_{4}=\langle R_{u}^{**},V_{w},PID_{u}^{new},SID_{d},T_{ta},T_{w}\rangle }{\to }$	
	
	
	
		$\;\mathrm{Check}\;\mathrm{if}\;\left|T_{w}-T_{c}\leq \delta T\right|$ $\;\mathrm{Select}\;R_{d}\in Z_{p}$$\;\mathrm{and}\;T_{d}$, $W_{t}^{*}=h\left(S_{GS}\left|\left|PID_{u}^{new}\right|\right|T_{ta}\right)),$ $V_{w}\overset{?}{=}h\left(SID_{d}\left|\left|W_{t}^{*}\right|\right|T_{w}\right),$ $R_{u}=R_{u}^{**}⊕h(S_{GS}||T_{ta})$, $SK_{du}=h\left(R_{u}\left|\left|R_{d}\right|\right|PID_{u}^{new}\left|\left|SID_{d}\right|\right|T_{d}\right)$, $R_{d}^{*}=R_{u}⊕R_{d}$, $V_{d}=h(SK_{du}||T_{d})$. $X_{d}=h(W_{t}^{*}||T_{d})$. $\overset{MSG_{5}=\langle V_{d},X_{d},R_{d}^{*},T_{d}\rangle }{\leftarrow }$	
	
	
	
	
	
	
	
		$\;\mathrm{Check}\;\mathrm{if}\;\left|T_{d}-T_{c}\leq \delta T\right|$, $\;X_{d}\overset{?}{=}h(W_{t}||T_{d})$ if true. $\;\mathrm{Select}\;\mathrm{timestamp}\;T_{w}^{*}$, $\;\overset{MSG_{6}=\langle V_{d},R_{d}^{*},T_{d},T_{w}^{*}\rangle }{\leftarrow }$	
	
	
$\;\mathrm{Check}\;\mathrm{if}\;\left|T_{w}^{*}-T_{c}\leq \delta T\right|$ $PID_{u}^{**}=PID_{u}^{*}⊕PID_{u}⊕PID_{u}^{new}$ $\mathrm{Replace}\;PID_{u}^{*}$$\;\mathrm{with}\;PID_{u}^{**}$ $R_{d}=R_{u}⊕R_{d}^{*}$, $SK_{ud}=h\left(R_{u}\left|\left|R_{d}\right|\right|PID_{u}^{new}\left|\left|SID_{d}\right|\right|T_{d}\right)$, $V_{d}\overset{?}{=}h(SK_{ud}||T_{d})$.			
$U\;and\;SD\;both\;Save\;\;the\;\;session-key\;\;SK_{ud}\left(=SK_{du}\right)$			

**Table 6:** Comparison of functionality features.

	[23]	[29]	[34]	[39]	[40]	[41]	Our
F_ua_	✓	×	✓	✓	✓	✓	✓
F_sna_	✓	×	✓	✓	✓	✓	✓
F_u_	✓	×	✓	✓	✓	✓	✓
F_para_	✓	✓	✓	✓	✓	✓	✓
F_sas_	✓	×	✓	✓	✓	✓	✓
F_ra_	✓	×	×	✓	×	×	✓
F_fopga_	✓	−	×	✓	×	×	✓
F_sia_	✓	−	×	✓	×	×	✓
F_epd_	×	✓	✓	✓	✓	✓	✓
F_rpca_	✓	−	×	✓	×	✓	✓
F_3fa_	✓	×	×	✓	×	×	✓
F_pska_	×	×	×	✓	×	✓	✓
F_sgvvn_	×	✓	✓	✓	✓	✓	✓
F_sva_	×	✓	✓	✓	✓	✓	✓
F_uia_	×	✓	✓	✓	✓	✓	✓
F_gwn_	×	✓	✓	✓	✓	✓	✓
F_sdi_	×	✓	✓	✓	✓	✓	✓
F_gwnia_	×	✓	✓	✓	✓	✓	✓
F_fasv_	✓	✓	✓	×	✓	✓	✓

Note: Fua: user anonymity; Fsna: sensor node anonymity; Fu: untraceability; Fpara: protection against reply attack; Fsas: secure against stolen smart-card attack; Fra: resilience against ESL attack under the CK-adversary model; Fopga: offline password guessing attack; Fsia: smart-card impersonation attack; Fepd: efficient protocol design; Frpca: resist password change attack; F3fa: three-factor authentication; Fpska: parallel session key attack; Fsgvvn: stolen GvVN attack; Fsva: stolen verifier attack; Fuia: user impersonation attack; Fgwn: GWN impersonation attack towards smart device; Fsdi: smart device impersonation; Fgwnia: GWN impersonation attack towards user; Ffasv: formal automated security verification.

**Table 7:** Communication cost comparison.

Protocol	# of Messages	# of Bytes
[23]	4	(68 + 40 + 56 + 72) = 236
[29]	5	(132 + 132 + 52 + 52 + 52) = 420
[34]	4	(132 + 64 + 40 + 68) = 304
[39]	8	(84 + 124 + 164 + 164) × 2 = 1072
[40]	3	(104 + 52 + 56) = 212
[41]	4	(100 + 52 + 52 + 84) = 288
Our	6	(76 + 112 + 16 + 72 + 60 + 44) = 380

**Table 8:** Approximate computation time of various operations.

Notation	Operation	Mobile Device	Gateway/TA	Smart Device
$T_{h}$	Hash function	0.009	0.004	0.006
$T_{ecm}$	ECC multiplication	5.116	0.926	4.107
$T_{sym}$	Symmetric enc/dec	0.017	0.008	0.013
$T_{bp}$	bilinear pairing	17.36	4.038	12.52

**Table 9:** Computation cost comparison.

Protocol	U	TA/RA	GWN	SD	Total Cost
[23]	$10T_{h}+1T_{fe}$	−	$10T_{h}$	$4T_{h}$	$24T_{h}+1T_{fe}$
	≈5.206 ms	−	≈0.04 ms	≈0.024 ms	≈5.27 ms
[34]	$6T_{h}+1T_{ecm}$	−	$7T_{h}+1T_{ecm}$	$3T_{h}$	$16T_{h}+3T_{ecm}$
	≈5.17 ms	−	≈5.114 ms	≈0.018 ms	≈10.302 ms
[39]	$7T_{h}+14T_{ecm}$	−	$12T_{h}+19T_{ecm}+4T_{bp}$	$7T_{h}+14T_{ecm}$	$26T_{h}+47T_{ecm}+4T_{bp}$
	≈71.687 ms	−	≈33.794 ms	≈57.54 ms	≈163.021 ms
[40]	$12T_{h}+3T_{sym}+2T_{ecm}$	−	$13T_{h}+4T_{sym}+2T_{ecm}$	$1T_{h}+1T_{sym}$	$26T_{h}+7T_{sym}+4T_{ecm}$
	≈10.391 ms	−	≈1.936 ms	≈0.019 ms	≈12.346 ms
[41]	$4T_{h}$	−	$5T_{h}$	$24T_{h}$	$33T_{h}$
	≈0.036 ms	−	≈0.02 ms	≈0.144 ms	≈0.2 ms
[29]	$4T_{h}+2T_{ecm}+3T_{sym}$	−	−	$3T_{h}+2T_{ecm}+3T_{sym}$	$7T_{h}+4T_{ecm}+6T_{sym}$
	≈10.319 ms	−	−	≈8.271 ms	≈18.59 ms
Our	$1T_{fe}+4T_{h}$	$1T_{sym}+4T_{h}$	$3T_{h}$	$6T_{h}$	$1T_{fe}+15T_{h}+1T_{sym}$
	5.152 ms	≈0.024 ms	≈0.012 ms	≈0.036 ms	≈5.224 ms

## Data Availability

Not applicable.
